# The time–temperature relationship for the inactivation of *Ascaris* eggs

**DOI:** 10.2166/washdev.2017.102

**Published:** 2017-12-05

**Authors:** D. Naidoo, G. L. Foutch

**Affiliations:** 1 School of Life Sciences, Westville Campus, University of KwaZulu-Natal, Durban 4051, South Africa; 2 School of Computing and Engineering, University of Missouri-Kansas City, Kansas City, MO 64110, USA and Chemical Engineering, Howard Campus, University of KwaZulu-Natal, Durban 4001, South Africa E-mail: foutch@okstate.edu

**Keywords:** *Ascaris sp.*, exposure time, inactivation, temperature, viscous heating

## Abstract

A time–temperature plot presenting the inactivation of *Ascaris* eggs is expanded with additional literature data. The information is of value to designers and operators of sanitation equipment who have *Ascaris* inactivation as an objective.

## INTRODUCTION

Lack of improved water, sanitation and hygiene (WASH) is associated with the infectious diseases that burden approximately one-third of the world’s population (Bardosh 2015). Access to WASH results in diarrhoeal disease prevalence, which manifests as a symptom of bacterial, viral and helminth infections – for the latter, the most common being *Ascaris* sp. (Fewtrell *et al*. 2005; Brownell & Nelson 2006).

Temperature is the most effective treatment option for sanitising human excreta and waste streams containing faeces (faecal sludge). Previous studies indicate that above 60_C *Ascaris* eggs are inactivated within a few minutes, but may survive more than a year at 40°C (Brownell & Nelson 2006). Viscous heating (VH) technology achieves high temperature quickly when a thick fluid passes through a narrow gap between a double cylinder with a stationary outer shell and a rotating inner cylinder (Belcher *et al*. 2015). The resulting shear field generates heat by molecular friction and inactivates *Ascaris* sp. eggs in faecal sludge (Podichetty *et al*. 2014). A key design variable of VH is the faecal sludge residence time at a specific operating temperature. Defining an effluent target temperature is an outcome of this communication. Previous studies, using both simulated and screened (sieved to remove debris) VIP (ventilated improved pit) latrine sludge, indicate that elevated temperatures (up to 95°C at atmospheric pressure) are achieved at low VH residence time (seconds) while deactivating helminth eggs (Belcher *et al*. 2015). Ensuring *Ascaris* is deactivated within this brief time is essential, and recent work (Naidoo 2017) defines high-temperature, low-exposure-time inactivation of *Ascaris*.

## SUMMARY OF THE EXISTING STUDY

Details of the experimental procedure and presentation of results can be found elsewhere (Naidoo 2017). Methodology is summarized briefly here. *Ascaris suum* eggs were procured and exposed to 60°C, 65°C, 70°C, 75°C and 80°C for 5, 10, 15, 30 and 45 seconds, and 1, 2, 3 and 4 minutes, respective to each temperature. Eggs were pipetted into plastic test tubes containing water, which had been preheated to the test temperature. Two samples (triplicated) were treated at each temperature/time combination and processed by either i) washing directly onto a 20 μmsieve (placed in a bowl containing tap water to allow for rapid cooling to room temperature), or ii) transferred into a beaker containing iced water (to allow for rapid cooling) and then washed onto the sieve. Eggs were immediately analysed via light microscopy, washed back into the test tube, and incubated for 28 days to determine whether further development occurred.

At 4-second exposure time, treatment at 80°C was sufficient, with <11% viable eggs recovered pre-incubation and <1% viable eggs recovered post-incubation. Eggs that appeared undeveloped but globular (indicating some form of morphological damage) did not develop further during incubation, indicating successful inactivation. Lower temperatures required longer exposure times (for example, treatment at 60°C required 3 or more minutes for visible damage), and from a visual examination of egg morphology the die-off mechanism appeared different.

## COMPARISON WITH LITERATURE DATA

**Figure 1:**
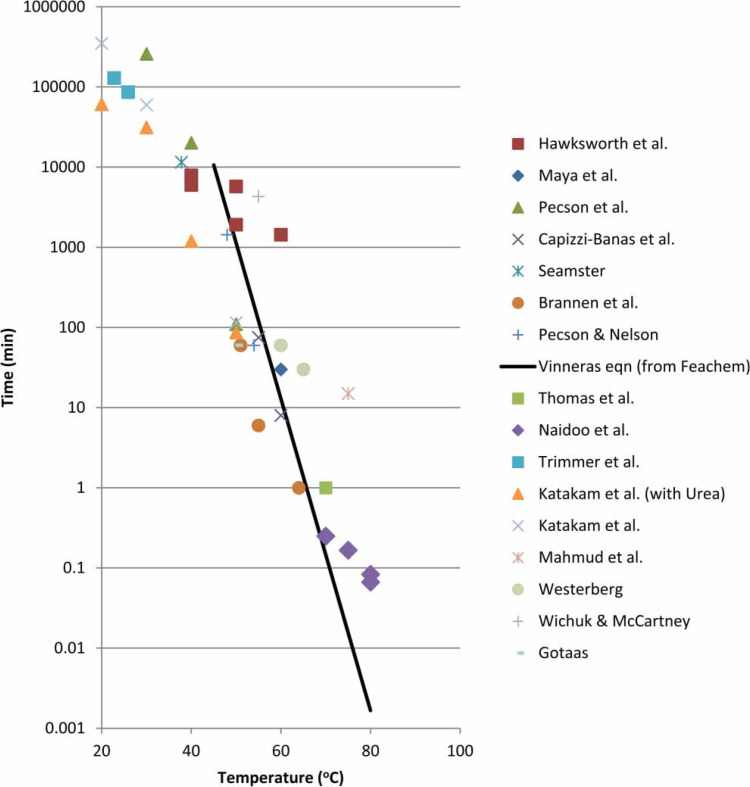
Comparison of time versus temperature inactivation data for *Ascaris* sp.

The time versus temperature plot of Thomas *et al*. (2015) presents comparative *Ascaris* inactivation data. An updated literature review found additional data for the Thomas *et al*. figure, as discussed below. The revised Figure 1 also includes data from Naidoo (2017) that extends the time–temperature range. References that cite inactivation as 99 + % are included. Experimental methods vary among the cited papers; a detailed comparative review is not presented.

Temperature is the focus of this data analysis, whether or not VH is used to generate the heat. Cited studies may include factors such as moisture content, alkaline concentration, pH and anaerobic operating conditions, but these are considered secondary influences and are not differentiated within the plot. The line in Figure 1 is that of Vinnerås *et al*. (2003) based on the data of Feachem *et al*. (1983). Their correlation begins at 45°C and does not extrapolate lower.

Data included are briefly discussed. Maya *et al*. (2012) concluded that times for both *A. lumbricoides* and *A. suum* showed negligible differences; as a result, Figure 1 includes both. Low temperature data of Kim *et al*. (2012), Berggren *et al*. (2004), Trimmer *et al*. (2016), Katakam *et al*. (2014) and Seamster (1950) are included in the plot. The latter considers the effects of chemical agents, and relative humidity (RH). Other researchers considered variables in addition to temperature: Hawksworth *et al*. (2010) included RH. Pecson *et al*. (2007) included pH. Capizzi- Banas *et al*. (2004) looked at lime and quick lime concentrations. Pecson & Nelson (2003) included pH and ammonia concentrations. Brannen *et al*. (1975) presented higher temperature data that included compost, water and faecal sludge with heat and/or radiation.

In examining the plot, the trend appears consistent with the Vinnerås *et al*. (2003) equation. A factor contributing to variability is that time scales may be overstated. For example, at high temperatures the Vinnerås *et al*. equation predicts inactivation of 0.1 sec at 80°C, while controlling exposure time in the laboratory is challenging at 1.0 sec. Below 45°C a new relationship may be appropriate.

Several studies are informative for practitioners but not presented in Figure 1 because complete inactivation was not reported. Vu-Van *et al*. (2016) monitored *A. lumbricoides* egg die-off over 181 days with average temperatures from 19 to 32°C and variables such as lime, rice husks and aeration. Berendes *et al*. (2015) studied inactivation at locations within pits with wide temperature and moisture content ranges. Fidjeland *et al*. (2015) developed an inactivation expression as a function of temperature and ammonia concentration up to 33°C. Yaya-Beas *et al*. (2016) presented inactivation percentages for an upflow anaerobic sludge blanket reactor operating at low temperatures. Manser *et al*. (2015) discussed inactivation near 35°C during anaerobic digestion and presented an inactivation model subsequently (Manser et al., 2016). Some data were omitted because the authors mentioned uncertainty or variability within the data (Brandon 1978; Steer & Windt 1978; Aitken et al., 2005; Popat et al., 2010).

## CONCLUSION

Based on data from the current study, 4–5 seconds of exposure at 80°C appears sufficient to inactivate *Ascaris* eggs. At 75°C and 70°C treatment may also be effective, but exposure time should be increased to achieve the same level of inactivation.
